# Dyslexia: Causes and Concomitant Impairments

**DOI:** 10.3390/brainsci13030472

**Published:** 2023-03-10

**Authors:** Reinhard Werth

**Affiliations:** Institute for Social Pediatrics and Adolescent Medicine, Ludwig-Maximilians-University of Munich, Haydnstr. 5, D-80336 München, Germany; r.werth@lrz.uni-muenchen.de; Tel.: +49-(0)1733550232

**Keywords:** dyslexia, reading impairment, causation, eye movements, simultaneous recognition, attention span, visual cortex, visual word form area

## Abstract

In recent decades, theories have been presented to explain the nature of dyslexia, but the causes of dyslexia remained unclear. Although the investigation of the causes of dyslexia presupposes a clear understanding of the concept of cause, such an understanding is missing. The present paper proposes the absence of at least one necessary condition or the absence of all sufficient conditions as causes for impaired reading. The causes of impaired reading include: an incorrect fixation location, too short a fixation time, the attempt to recognize too many letters simultaneously, too large saccade amplitudes, and too short verbal reaction times. It is assumed that a longer required fixation time in dyslexic readers results from a functional impairment of areas V1, V2, and V3 that require more time to complete temporal summation. These areas and areas that receive input from them, such as the fusiform gyrus, are assumed to be impaired in their ability to simultaneously process a string of letters. When these impairments are compensated by a new reading strategy, reading ability improves immediately.

## 1. Introduction

Since the German ophthalmologist Oswald Berkhan [1] first described the symptoms of dyslexia in 1881 and Rudolf Berlin introduced the term ”dyslexia“ [2], numerous theories have been proposed about its causes and treatments, [3,4] (for review). The magnocellular theory of dyslexia [5,6,7,8,9], the theory of unusual foveal and parafoveal processing of letters including an unusual crowding effect [10,11,12,13,14,15,16,17,18,19], and the temporal summation theory [20,21,22,23] regard developmental dyslexia (DD) as a visual perceptual disorder. Other theories assume that DD results from an impaired ability to process auditory stimuli [24,25,26,27] or is caused by impaired control of reading eye movements [6,7,8,9,28,29,30,31,32,33,34,35,36,37,38,39,40,41,42,43].

Although the phonological awareness theory of DD has the most followers, it cannot adequately explain what causes DD. This theory includes different abilities, such as identifying phonemes; rhyming; naming letters, objects, and colors, and splitting words into syllables. It is assumed that an impairment in these abilities causes DD and that DD is due to an impaired ability to associate letter sequences with sound sequences [44,45,46,47,48,49,50,51,52,53,54,55,56]. Such impairments may coexist with DD, but a causal relationship between these impairments and DD has never been proven. Studies on the correlation between various performance deficits and dyslexia assume that deficits in the phonological domain are most frequent in readers with dyslexia, whereas deficits in the visual domain seldom occur [57]. However, this result is predetermined by the study design because visual influences, such as fixation location in the word, fixation time, direction and amplitudes of reading saccades, and extent of the visual field of attention, have not been examined (e.g., [57]). Assessing the impact of these visual influences proved them to be necessary conditions for reading, and their absence caused impaired reading [20,21,22].

Noncausal correlations between impaired reading performance and other performance impairments must be clearly distinguished from causal relationships. A causal relationship can only be stated when the criteria for a causal relationship have been clarified. A major weakness of theories concerning DD is that research in DD has hitherto ignored the longstanding discussions in the philosophy of science attempting to specify these criteria, presumably because previous mathematically formulated approaches [58,59,60,61,62,63,64,65] are difficult to transfer to experimental research. Therefore, the present study aims to formulate simple experimental criteria for a causal relationship as opposed to correlations without a causal relationship. An impairment can only be considered a cause of poor reading ability if there is a clear and logically consistent concept of ‘cause’ which can be applied to the search for causes of dyslexia. Since the concept of cause is based on the concepts of necessary and sufficient conditions [66,67,68,69,70,71,72,73], these concepts must be defined in terms of experimental methods. The definition provided here does not use a mathematical approach but offers a description of the notion of causality based on the experimental practice. The use of this approach can identify necessary and sufficient conditions in the study of dyslexia.

## 2. What Are Causes of Dyslexia?

### 2.1. Necessary Conditions, Sufficient Conditions, and Causes

A condition is considered necessary if it is indispensable for correct reading even if at least one sufficient condition is fulfilled. Suppose it can be demonstrated in a statistically sufficient number of experimental trials that pseudowords made up of five letters can be read correctly if the fixation time is at least 500 ms and that they cannot be read correctly if the fixation time is less than 500 ms. Then a fixation time of at least 500 ms is a necessary condition for five-letter pseudowords to be read correctly, provided all other experimental conditions remain constant.

A condition is considered sufficient for correct reading if it is dispensable when at least one other sufficient condition and all necessary conditions are fulfilled. Suppose it can be demonstrated in a statistically sufficient number of experimental trials that pseudowords can be read correctly if they are made up of four letters and if the fixation time is at least 500 ms. Let us also assume that pseudowords cannot be read correctly if they are composed of more than four letters or if the fixation interval is less than 500 ms. This implies that a word length not exceeding four letters is a sufficient (but not necessary) condition for pseudowords to be read correctly, and a fixation time of at least 500 ms is a sufficient (but not necessary) condition for pseudowords to be read correctly. To read words correctly, only one of these two conditions needs to be met, and each condition can be swapped with the other.

Def. 1: Let D be the set of all conditions under which an event E occurs (e.g., a person P can read flawlessly). Elements of D may exist at the same time as E or may have existed before E. Let N be a subset of D that contains only the conditions N_1_, …, N_k_, and let H be a different subset of D that contains only the conditions H_1_, …, H_q_. Then an element of N is a necessary condition for E (flawless reading) if and only if E is no longer present (flawless reading is no longer possible) if at least one element of N is missing (or has been missing) and at least one element of H is (or was) present.

An element (condition) of H is a sufficient condition for an event E (flawless reading) if and only if E is present (flawless reading is possible) if this element of H or other elements of H are present (or were present) and all elements of N (which are different from the conditions that are elements of H) are (or were) present. E is not present (a flawless reading is no longer possible) if all conditions that are elements of H are missing even if all elements of N are (or were) present.

In addition to the elements of sets N and H, another set of conditions (C) must be fulfilled. Only if conditions that are elements of C are fulfilled, conditions that are elements of the set N become necessary, and conditions that are elements of the set H become sufficient. For example, in the normally developed brain, the fibers from the nasal halves of the retinae cross in the optic chiasm so that the information reaches the contralateral cerebral hemisphere. In contrast, the fibers from the temporal halves of the retinae reach the ipsilateral cerebral hemisphere. Thus, stimuli on the right halves of the retinae are processed in the right cerebral hemisphere. These anatomical conditions (conditions which are elements of set C) are the presuppositions that a functional right occipital lobe is a necessary condition for visual stimuli to be detected when they are projected onto the right halves of the retinae (i.e., when they appear in the left visual hemifield). However, if unusual neuronal connections have developed after surgical removal of the right hemisphere in early childhood, connecting the entire retina with the remaining left hemisphere, the existence of a functional right occipital lobe is no longer a necessary condition for the processing of visual stimuli in the left visual hemifield.

There are many necessary and sufficient conditions for a person to be able to read correctly, including anatomical, physiological, biochemical, and psychological conditions. These conditions cannot all be explicitly formulated and are only partially known to the examiner. According to Definition 1, if a person is able to read, all the necessary conditions and at least one sufficient condition for reading are fulfilled, although we do not know them all. Nevertheless, we are able to find out which necessary condition is missing or if no sufficient condition is fulfilled. Since there are many necessary and sufficient conditions, more than one necessary condition may be missing, many sufficient conditions may be missing and may not have been replaced by other sufficient conditions, or even all sufficient conditions may be missing. This means that many necessary conditions and/or many unreplaced sufficient conditions may be missing. Therefore, dyslexia may not always have a single cause, but may have many causes. To recognize the necessary and sufficient conditions for better reading, reading performance must be tested in the alternating presence and absence of conditions suspected to be necessary or sufficient for reading. This means that we need to examine the conditions under which readers make reading errors and the conditions under which the same readers read correctly. To demonstrate the influence of conditions on reading performance, it is necessary to compare a group of subjects whose reading performance is tested in the presence and absence of these conditions (elements of D) with a control group. The reading performance of the control group is tested under unchanged conditions. 

If it is demonstrated that reading performance is reduced or even impossible because at least one or more necessary conditions are lacking and/or because no sufficient condition is present, these are causes of reduced reading performance or the inability to read. Concerning the concepts of causation specified here and earlier [58,59,60,61,62,63,64,65], impairments that have been demonstrated to occur together with DD (e.g., [13,24,25,26,27,28,29,30,31,32,33,34,35,36,37,38,39,40,41,42,43,44,45,46,47,48,49,50,51,52,53,54,55,56,57,74,75,76,77,78,79,80,81,82,83,84,85,86,87]) turn out to be only concomitant impairments that do not fulfill the requirement for a causal relationship.

It may be that conditions that appear to be necessary or sufficient conditions are composed of several features, but only one feature may be a necessary or sufficient for reading ability whereas other features may have no influence. In this case, not all features that appear to constitute the necessary conditions can be regarded as necessary, and not all features that constitute the sufficient conditions can be regarded as sufficient. Then it must be investigated experimentally which features are necessary or sufficient and which features are irrelevant. If one finds for example that it is a necessary condition for a person to be able to read words when they are presented with a luminance of 4 cd/m^2^ on a 68 cd/m^2^ background, then only the difference in luminance is a necessary condition for reading. The fact that the words are presented in dark blue or in black is an irrelavant feature. The color is not part of the necessary condition.

Poor reading ability can result from many different impairments. The problem is to rule out all possible causes of dyslexia other than those being investigated. Many possible causes are evident because they can be easily identified and ruled out. Examples include eye diseases that prevent a clear image of the word to be projected onto the retina, visual field defects, or amblyopia. They are easy to diagnose. Other possible influences, such as inappropriate eye movements, incorrect fixation of the word to be read, too short a fixation time, insufficient focus of attention, the number of letters that can be recognized simultaneously (simultaneous recognition), and the verbal reaction time that a reader needs to retrieve sound sequences from memory when reading aloud, are not usually assessed in routine reading tests. The mere finding that these possible influences are associated with reading difficulties does not allow us to conclude that they have a causal influence on reading performance.

If it can be proven that normal readers and dyslexic readers only differ in one feature F_1_ which is present in good readers and absent in poor readers, it may be concluded that the absence of feature F_1_ is the cause of dyslexia. Thus, it is assumed that the presence of F_1_ is a necessary condition for good reading and its absence is a cause of dyslexia. This conclusion is correct only if all other possible causes have been ruled out and if it has been demonstrated that the presence of F_1_ is indeed a necessary condition for good reading. To test whether a possible influence (F_1_) is a necessary condition for normal reading ability, the only way to do this is to test whether reading normalizes when F_1_ is present and whether reading deteriorates when F_1_ is absent. However, this is not possible in many cases. A blind or amblyopic area of the retina cannot be made to disappear or reappear at will. In this case, knowledge of the visual system may lead to the conclusion that reading is not possible if the foveal and perifoveal areas of the retina are blind or severely amblyopic. According to Def. 1, such a conclusion must be based on the assumption that visual function would return if the damaged areas of the visual system recovered and regained their function. This hypothesis is supported by the observation that visual function can recover in previously blind areas of the retina after the function of neural networks in an affected region of the visual system has been restored.

As mentioned above, the question of whether fixation duration is a necessary condition for reading can be easily tested by offering pseudowords with different presentation times. However, prolonging fixation time alone may not improve the ability to recognize pseudowords. Using pseudowords instead of natural words has the advantage that pseudowords can only be read correctly if every letter is recognized. A natural word can be correctly guessed if only a few letters and the shape of the word are recognized. The ability to recognize pseudowords depends on the length of the fixation time and the number of letters that make up a pseudoword. It is therefore necessary to manipulate both simultaneously in order to test whether both are sufficient conditions for pseudoword recognition. There are significant differences between readers in the number of letters that can be recognized simultaneously. Repeated studies [20,21,22] have shown that good readers can often recognize at least six letters simultaneously in less than 250 ms, whereas some poor readers require a fixation time of at least 500 ms to recognize three letters. As fixation time increases, an increased number of letters can be recognized simultaneously. Reading errors occur when children try to recognize more letters at the same time than they can. Letters are then omitted, replaced by other letters, moved to the wrong place in the word, or letters are added to the word that are not in the word being read [20,21,22].

Even if the number of letters in the pseudowords is limited (e.g., to four letters) and the fixation time is sufficiently prolonged (e.g., up to 500 ms), many subjects may still not be able to recognize all the pseudowords correctly. It has been shown that subjects are only able to do this if the verbal reaction time during reading aloud is sufficiently prolonged (on average to approximately 1500 ms). This was achieved by offering a sound after the presentation of the pseudoword and instructing the subjects to begin pronouncing the pseudoword to be read only after the sound. The time between the pseudoword presentation and the start of the correct pronunciation of the pseudoword was measured using a computer. Under appropriate conditions, even children with severe dyslexia were able to correctly read at least 95% of the pseudowords [20,21,22].

### 2.2. Dyslexia Is Not Always Due to an Impaired Visual Attention Span, to Lateral Masking, or a Phononological Impairment

The visual attention span hypothesis assumes that the ability to process multiple letters that make up a word is impaired in children with dyslexia and that this is due to a reduced visual attention span [88,89,90,91,92,93,94,95,96,97,98,99,100,101,102,103,104,105,106,107,108]. Whether this impairment is due to a reduced attention span or any other visual deficit depends on what is understood by ”attention span“. Bosse et al. [90] (Abstract) defined ”… the visual attentional span is the amount of distinct visual elements which can be processed in parallel in a multi-element array“. The question is whether this definition is appropriate and how visual attention can be distinguished from other visual performances.

Def. 2: Visual attention is directed to a location in the visual field under given environmental conditions:

(1) If the processing of stimuli improves when a person voluntarily attempts to process visual stimuli optimally at that location;
(2) and/or if the processing of stimuli improves when stimuli are (consciously) expected at that location;
(3) and/or if the processing of stimuli improves when environmental conditions indicate the location where stimuli will appear;
(4) and/or when the visual system attempts to process stimuli optimally at that location even if the person does not voluntarily try to process stimuli optimally at that location.

The finding that dyslexic readers perform worse than normal readers on visual attention tasks [90,91,92,93,94,95,96,97,98,101,102,103,104,105,106,107,108] does not allow us to conclude that this reduced visual attention causes dyslexia. Poor visual attention revealed by attention tests may only accompany dyslexia but not cause it. The question remains whether the poorer performance of dyslexic readers is due to an attentional or a sensory deficit [99,100]. Confirming a causal relationship between poor attention and dyslexia requires normalizing attention in dyslexic readers and testing their reading performance under normal and impaired attention capacities. If this is not possible, the role of attention in reading must be inferred from readers’ performances on reading tests. Dyslexic readers have longer verbal reaction times than normal readers [101,102,103] and perform worse than normal readers when reading five-letter strings presented for 200 ms [90]. They also need longer exposure times than normal readers when reading single letters [107]. Dyslexic children performed worse than normal-reading children when requested to pick a string of symbols that had been previously shown for 100 ms from two symbol strings. The authors concluded that dyslexic readers had poorer visual sensitivity than normal readers and that there is an “… hitherto unrecognized visual component in children’s reading difficulties” [108].

When examining a person´s ability to read a three-letter pseudoword as well as s/he can, a mark can be presented on a monitor, and the person can be asked to fixate on this mark. A pseudoword to be read can then be displayed on the monitor such that the middle of the pseudoword matches the location of the fixation point. The pseudoword to be read is then located in the fovea, the location with the highest visual acuity. Simultaneously, all distracting stimuli must be eliminated. It has been demonstrated that many children with dyslexia are even unable to recognize three letters within a fixation time of 250 ms under these experimental conditions. When the fixation time was prolonged up to 500 ms, all children (n = 200) were able to recognize three letters simultaneously [20,21,22]. This shows that the children were able to focus their attention on the words when given sufficient time. The result can be interpreted in terms of attention as items (1)–(3) of Def. 2 are fulfilled. The number of letters in pseudowords that could be read without error also depended on the fixation time for four-letter, five-letter, and six-letter pseudowords. According to the definition provided by Bosse et al. [90], this results from a reduced attention span. However, this was not the case. If the children were unable to recognize pseudowords that were presented for 250 ms after the children had focused their attention for several seconds on the fixation point and a pseudoword was subsequently presented, the children focused their attention for some seconds plus 250 ms on the location where the pseudoword appeared. This fixation time was not sufficient to recognize the pseudoword. However, if pseudowords were always correctly recognized at a presentation time of, e.g., 500 ms, the few seconds in which attention was focused on the fixation point plus a pseudoword fixation time of 500 ms was sufficient to correctly recognize almost all letters in the pseudowords. The difference in 250 ms fixation time was decisive. The time interval in which the children focused their attention on the fixation point before a pseudoword appeared varied from trial to trial. However, the inability to recognize a pseudoword presented for 250 ms was independent of the time children focused their attention on the fixation point before a pseudoword appeared. This means that the time given to the children to focus their attention on the location where the pseudoword appeared did not influence their ability to recognize the pseudoword. Recognition of the pseudoword was determined solely by the time interval during which the pseudoword appeared. This means that the children did not need more time to focus their attention on the location where a pseudoword appeared. The children were also able to extend their attention to the entire pseudoword because at a longer presentation time, all pseudowords could be recognized. According to Def. 2, an impaired ability to recognize a given number of letters that make up a pseudoword within a sufficiently long period of fixation cannot result from a reduced attention span.

Vidyasagar and Pammer [109] tried to explain dyslexia as a deficit in visuo-spatial attention due to poor control of the dorsal visual pathway. The authors assume that “… letters are recognized sequentially with only one or a few letters being processed at a time by the object recognition system and this temporal sequence preserves the special sequence of letters” ([109] p. 58). They assumed that single letters or small groups of letters are scanned sequentially from left to right at a speed of less than 45 ms per item during fixation of a word. This shift in attention is believed to be controlled by the mainly magnocellular pathway of the dorsal stream including the posterior parietal cortex. Neurons in the dorsal stream are assumed to provide information about the location of items to be selected for detailed sequential analysis and recognition by neurons of the ventral stream. Dyslexia is believed to be caused by a dysfunction of magnocellular neurons in the dorsal pathway. According to this theory, a dyslexic reader who can recognize only two or three letters at a time attempts to read a six-letter word moving his/her focus of attention in steps of only two or three letters from left to right over the word, reading a sequence of only two or three letters at a time. Thus, dyslexic readers should be able to read words of any length that are in the area of sufficient visual acuity. However, this is not the case. If the theory were correct, a child who can read three letters at a time should always be able to recognize the first three letters of a word correctly, regardless of its length. However, dyslexic readers who can read three-letter words perfectly often misread the first three letters of five- or six-letter words and are unable to recognize letters at any position in the word [20,21,22]. The recognition of letters at the beginning of the word is also affected by the length of the whole word. This would not be the case if a reader were to read by recognizing a sequence of segments, one after another, each consisting of only two or three letters. The theory also fails to explain why the frequency of reading errors increases from the first letter at the beginning of the word to the third and remains approximately constant for the fourth, fifth, and sixth letters in words longer than three letters [20,21,22]. The theory implies that the ability to read two or three letters at a time decreases from left to right. This also remains unexplained.

Dyslexia can be regarded as the result of the longer fixation time needed to complete temporal summation. This is in agreement with the finding that detection and recognition of visual stimuli and visual acuity improve with an increase in the fixation interval, i.e., with increasing temporal summation [110,111,112,113,114,115,116,117,118,119,120,121]. These results are also in agreement with the finding that responses of the visual cortex increase monotonically but sub-linearly with increased duration of the stimulus [119,120,121,122,123,124]. An increase in visual fixation time results in an increase in the time interval during which temporal summation is completed. Temporal summation has been demonstrated predominantly in areas V1, V2, and V3 and to a lesser extent in areas V4, the anteriorly adjacent area VO, the occipitotemporal cortex corresponding to area MT, and the intraparietal sulcus [119,125]. The finding that the ability to read pseudowords immediately improved to the extent that all dyslexic readers could correctly recognize at least 95% of pseudowords of a given length when the fixation time was increased [20,21,22] shows that there is not only a correlation but also a causal relationship between reduced reading performance and the time available for temporal summation and recognition. Children with DD need more time for temporal summation and visual recognition.

Many dyslexic readers are unable to recognize all the letters in a pseudoword consisting of more than three or four letters, even if the subjects´ gaze is directed to the center of the word in the absence of distracting stimuli, when the subject is given sufficient time to focus his/her attention on the word and when the subject makes every effort to do so. These readers’ reading performance immediately improved when the number of letters to be recognized simultaneously was reduced [20,21,22]. The assumption that impaired simultaneous recognition in reading is due to early cortical processing is supported by the finding that the numbers of objects that are visually processed at a time without counting them is a basic feature of the early stages of processing in the visual system. Event-related potentials (ERP) have shown that neural responses approximately 90 ms after stimulus onset are sensitive to the number of items to be registered simultaneously [126]. The intensity of the BOLD signal in functional MRI increased in areas V1, V2, and V3 when the number of items to be registered in a visual array increased [127]. Longer words activated the medial and superior lingual gyrus, fusiform gyrus, and medial cuneus [126,127,128,129]. These results indicate that DD is due to an impairment of the visual system that requires longer fixation times and that has a decreased ability to recognize multiple letters at a time [20,21,22]. 

An inability to recognize several items at a time (simultaneous agnosia) has already been reported in brain damaged patients [130,131,132,133,134,135,136,137,138,139,140]. These patients were unable to overview a set of objects in an otherwise unimpaired visual field. They could only detect one object among several ones at a time, although there was no visual field defect when the visual field was assessed with a single stimulus. Impaired simultaneous recognition in reading may be regarded as a mild form of simultaneous agnosia that only becomes apparent in tasks such as reading. An area in which only a limited number of letters can be recognized at a time is different from a visual field of attention. The visual field of attention is an area in the retina where the detection and recognition of visual stimuli is improved and which expands according to how much attention is focused on this area [141,142,143,144,145]. In contrast, an area in which only a limited number of letters can be recognized cannot be expanded nor can more letters be recognized even if all attention is focused on that area.

It has also been assumed that dyslexia may be due to an unusual masking of letters by flanking letters (crowding effect) [10,11,12,13,14,15,16,17,18,19]. In this case, letters to the left and to the right of a letter decrease its recognition. Recent studies have shown that crowding plays no decisive role in the recognition of a letter string consisting of up to six letters. Letters at the end of pseudowords flanked by a letter on only one side were misread as often as letters flanked by letters on both sides [20,21,22]. The results of these studies demonstrate that the rate of incorrectly read letters in three-, four-, and five-letter pseudowords increases from the first letter up to the third letter but not from third to fifth letter. In six-letter pseudowords, the rate of misread letters increased only from the first to the fourth letter and remained constant from the fourth to the sixth letter. The fixation point was at the third letter in all trials. All dyslexic readers were able to read at least 95% of the pseudowords correctly when they fixated them in the middle, when the length of the pseudowords was adjusted to the children’s ability to recognize a string of letters at a time, and when the fixation time and the time from the beginning of the presentation of the word to the start of pronouncing the word was sufficiently prolonged. Whenever a word was read incorrectly, it was subsequently also spelled incorrectly [20,21,22]. This demonstrates that words were misread because the letter strings were not visually recognized correctly and that the crowding effect played no important role.

The finding that dyslexic readers were able to recognize and pronounce 95% of the pseudowords correctly when sufficient fixation time and enough vocal reaction time were provided demonstrates that all dyslexic children were able to associate the correct sequence of sounds with the sequence of letters [20,21,22]. Therefore, DD cannot generally be attributed to an impaired ability to associate letter sequences with sound sequences, as hypothesized by the phonological awareness theory [44,45,46,47,48,49,50,51,52,53,54,55,56].

When reading a text, a sufficiently long fixation period, a limit on the number of letters a subject tries to read at a time, and a sufficiently long verbal reaction time when reading aloud may not yet significantly improve reading performance. This raises the question of what role eye movements play in reading, whether normal reading eye movements are a necessary condition for normal reading, and whether unusual eye movements are a cause of reading difficulties. Findings on the role of fixation times, the number of letters a reader can recognize simultaneously, and the role of verbal reaction time already show that reading difficulties cannot be attributed solely to unusual eye movements during reading. The role of reading eye movements in dyslexia needs to be assessed in relation to each reader’s ability to recognize a string of letters simultaneously within a given fixation interval and in relation to the verbal reaction time required by each reader.

### 2.3. The Role of Inappropriate Reading Eye Movements in Dyslexia

The question of whether reading eye movements that deviate from the norm can cause a ‘‘reading disorder’’ has been controversial [6,7,8,9,28,29,30,31,32,33,34,35,36,37,38,39,40,41,42,43]. It has been argued that irregular eye movements often found in subjects with a ‘‘reading disorder’’ can also occur in good readers and that some poor readers also demonstrate normal eye movements.

Reading requires that the word or word segment to be read is displayed in the area of the retina that has a sufficiently high visual acuity: the fovea and perifoveal area. This is true for all languages. To make the best possible use of the highest visual acuity area, the center of the word or word segment to be read should be located at about the center of the fovea. When the reader directs his/her gaze toward the beginning of a word or word segment to be read, the word or word segment to be read is shifted to the right half of the fovea and perifoveal area, and letters at the right end of the word or word segment to be read may be outside the range of sufficiently high visual acuity, and they cannot be recognized. When the reader directs the gaze toward the end of the word or word segment, the word or word segment is shifted to the left half of the fovea and perifoveal area. The letters at the beginning of the word or word segment may then be outside the range of sufficiently high visual acuity and cannot be recognized. This means that the position of the gaze in the word is a necessary condition for simultaneously recognizing as many letters as possible.

**Figure 1:**
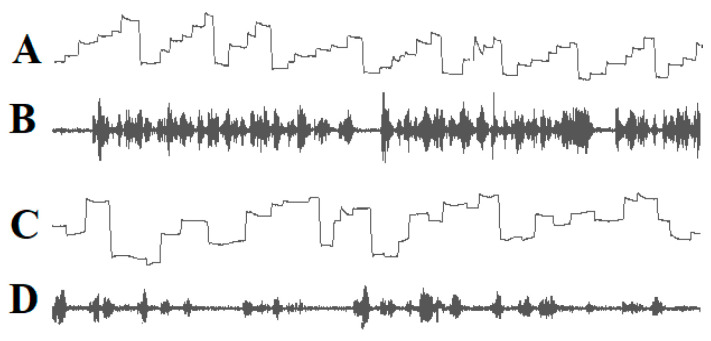
Eye movements (**A**) and speech spectrogram (**B**) of a good reader. Ascending lines: eye movements to the right; descending lines: eye movements to the left. The reader exerts a sequence of staircase-like eye movements in the reading direction. The speech spectrogram shows fluent reading without interruptions. Irregular eye movements (**C**) and the speech spectrogram (**D**) of a dyslexic child. The child exerts many eye movements opposite to the reading direction. The speech spectrogram demonstrates that the child reads haltingly with many pauses.

Since the words or word segments to be read must be shifted into the fovea and perifoveal area, the eyes must move in the reading direction, and the saccade amplitude must be adjusted so that after each saccade, the next word to be read is projected onto the fovea and perifoveal region. Good readers complete a succession of staircase-like reading saccades in the reading direction whereas many poor readers execute irregular eye movements that are often directed opposite to the reading direction (Figure 1).

If we compare good readers with poor readers and find that poor readers make more irregular eye movements than good readers, we may assume that there is a causal relationship between irregular eye movements and poor reading performance. The assumption of a causal relationship implies that poor reading performance disappears when eye movements are normalized. If reading performance does not improve after eye movements have normalized, a non-causal correlation must be assumed. This means that it must be examined whether abnormal eye movements are a necessary or sufficient condition for poor reading performance. The normalization of eye movements can be achieved by guiding reading eye movements using a computer. It can be tested whether saccades of appropriate amplitudes, a sufficiently long fixation interval, a sufficiently long verbal reaction time, and an attempt to recognize no more letters than a reader can, lead to a drastic reduction in reading errors.

In our experiments [20,21,22,146,147]:

(1) A yellow cursor indicated the location in the word where the gaze should be directed;
(2) Green cursors to the left and right of the yellow cursor indicated how many letters to the left and right of the fixation point the subject should try to recognize together with the letter at the fixation point;
(3) The onset of the movement of the yellow and green cursors in the reading direction indicated when fixation on a word or word segment should be terminated;
(4) The amplitude of the movement of the cursors in the reading direction indicated the direction and amplitude of the saccade to be made in order to read the next word or word segment;
(5) A sound signal indicated when the subject should begin to pronounce the word or word segment to be read.

Five independent studies in which 350 children with dyslexia participated [20,21,22,146,147] have shown that reading errors decrease immediately with an effect size of up to Hedges g = 2.65 if:

(1) Fixation times are long enough to recognize a string of letters;
(2) Readers do not try to recognize more letters in a word at the same time than they can;
(3) Eye movement amplitudes in the reading direction do not exceed the number of letters that the reader can simultaneously recognize;
(4) The reader does not start pronouncing the words or word segments to be read too early.

**Figure 2:**
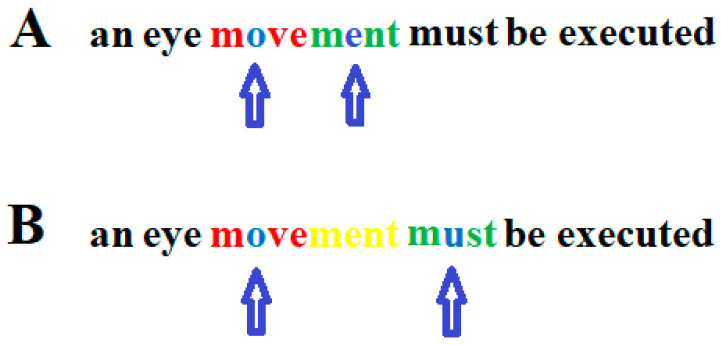
Correct (**A**) and too large (**B**) saccade during reading. In the upper graph, the arrows indicate on which letter within a word segment a person who can only recognize four letters simultaneously must focus his/her gaze. One letter to the left and two letters to the right (red) of the focused letter (blue) must be recognized simultaneously together with the focused letter. After the first word segment (blue and red) has been read, the gaze only moves so far in the reading direction that the next word segment to be read (blue and green) follows without a gap between the two word segments. (**B**): After the first word segment (blue and red) was read, the eyes jumped too far to the next word segment (blue and green) so that the letters “ment” (yellow) were overlooked. Arrows indicate the letter on which the gaze is focused.

Eye movements were recorded to check whether the readers´ eyes followed the curser. If the reader’s eye movements did not follow the cursor exactly, the text to the left and right of the word being read was erased. In this way, staircase-like eye movements can be induced, such as those seen in good readers. However, inducing staircase eye movements in dyslexic readers, such as those seen in good readers, is not sufficient to improve the reading ability in dyslexic readers. Whether reading eye movements are appropriate does not depend on whether the sequence of saccades matches that of good readers. Rather, it depends on the individual’s ability to simultaneously recognize a string of letters and the duration of the fixation interval required to detect a given string of letters that make up a word [20,21,22,146,147]. Therefore, eye movements cannot be considered a cause of dyslexia if they are unusual and deviate from the norm represented by typical readers. After a word or word segment has been read, a saccade is initiated to the next word or word segment to be read. This saccade should be aimed at approximately the middle of the word or word segment to be read subsequently. The amplitude of the saccade must not exceed the number of letters that can be recognized simultaneously so that the word segment to be read next follows the previous word segment without a gap between the word segments (Figure 2).

When a five-letter word segment has been read with the gaze directed to the middle letter and a saccade over seven letters to the next word segment has been executed, the child can recognize the fixated letter and two letters to the left and two letters to the right of the fixated middle letter of the word segment to be read. If the child can only recognize five letters at a time and such a large saccade is executed, two letters are overlooked between the two word segments. This demonstrates that the amplitudes of reading saccades that exceed the number of letters that the reader can recognize simultaneously will cause reading errors. Thus, whether the amplitude of a reading eye movement is appropriate depends on the number of letters a reader can simultaneously recognize. As the duration of the fixation interval also determines whether a word can be correctly recognized, the time at which a saccade is initiated is critical for flawless reading.

To execute adequate eye movements that match the number of letters that can be recognized simultaneously, the reader must be able to control reading eye movements. It has been demonstrated that even the eyes of children with severe dyslexia can be guided by a computer so that they can learn to execute appropriate reading eye movements within fewer than 30 min [20,21,22,146,147]. This shows that the children were able to control their eye movements but did not do so spontaneously and had to control them voluntarily. This means that reading disorders do not occur because eye movements deviate from the norm but because the amplitudes of many eye movements are too large and do not correspond to the length of simultaneously recognized words, which causes letters to be overlooked (Figure 2).

When children meet the requirements for flawless reading, appropriate saccades may be interspersed with eye movements opposite to the reading direction or with searching eye movements in both directions. As long as there is a sequence of correct saccades, unusual saccades that occur between the correct saccades do not make flawless reading impossible. As long as readers fixate the words to be read for a sufficiently long time, do not try to recognize more letters than they can at a time, do not start pronouncing the words to be read too early, and make appropriate saccades, eye movement impairment does not result in reading impairment.

If a reader´s fixation time is too short and/or if a reader tries to recognize more letters simultaneously than s/he can, many words will not be recognized. Readers realize that the words they attempted to recognize were incorrectly recognized and that a sequence of words does not make sense. Then, readers often make eye movements opposite to the reading direction to refixate on words that have already been read or make searching eye movements in and opposite to the reading direction [21,40]. In this case, unusual eye movements are the result of reading impairments such as too short fixation times and/or the inability to recognize as many letters simultaneously as the reader tries to recognize. Thus, unusual eye movements can be a cause as well as a consequence of poor reading performance.

### 2.4. DD Is Not Caused by a Phonological Impairment

Reading requires knowing which sounds are associated with which letters or sequences of letters. In languages such as Spanish, German, and Italian, where there is a high grapheme–phoneme correspondence, it is easier to learn the association between sounds and letters than in languages such as English or French in which the pronunciation of the same letter can vary in different words and a sound is often represented by a sequence of letters. The ability to learn the grapheme–phoneme association of a language is not equally developed among all individuals. Even some children with German as their native language have great difficulty learning the grapheme–phoneme correspondence of certain letters, such as “m/n, b/d, or p/q”. Therefore, it is not surprising that children whose native languages have ambiguous grapheme–phoneme correspondence have even greater difficulties. These problems can be easily eliminated in the case of native German speakers by testing whether the grapheme–phoneme connection is mastered for all letters and letter sequences and excluding those children who do not know the phonemes associated with certain letters. The causes of reading problems can then be examined independently of the inability to store the association between some letters and the corresponding sounds in memory. In languages where grapheme–phoneme correspondence is variable and sophisticated, readers must retrieve pronunciation from memory when reading real words or pseudowords. This problem is present in all reading tests with native English and French speakers. Even in three-letter words (e.g., the pronunciation of the letter “a” in the words ‘tar’, ‘raw’, ‘tan’ or the pronunciation of the letter “e” in words like “sea”, “set”, “sew”), the pronunciation of letters can vary. In languages such as Spanish, German, or Italian, the problem of variable pronunciation of letters is almost non-existent. In these languages, the causes of reading problems can be investigated independent of the impaired ability to master the grapheme–phoneme correspondence. It has been demonstrated that children may have severe DD even if there is no impairment in their knowledge of grapheme–phoneme correspondence. Studies with native German speakers in whom impaired knowledge of grapheme–phoneme correspondence was excluded [20,21,22,146,147] demonstrated that impaired knowledge of grapheme–phoneme correspondence plays only a minor role in dyslexia. The causes of DD revealed in native German speakers will also be present in dyslexic English or French readers because the necessary conditions for reading German are the same as for reading English or French. If native English or French speakers have reading problems due to impaired knowledge of grapheme–phoneme correspondence, they may not be dyslexic in a language with high grapheme–phoneme correspondence. Since dyslexia can have causes other than impairment in finding the correct sequence of sounds for a sequence of letters, other possible causes must be examined independently of a phonological impairment. From the definition of “cause” given above, it follows that any study that aims to find causes of dyslexia must be a study involving therapy because it investigates whether the presence or absence of a condition improves or worsens the reading ability of a dyslexic reader.

## 3. Is Dyslexia Due to an Impaired Learning Capacity?

It may be assumed that dyslexia develops because children cannot learn to read due to a reduced learning capacity. This assumption is supported by studies which have found that dyslexic readers perform worse than normal readers on tasks such as the digit span test [148]. Children with dyslexia also showed significantly poorer performance than normal-reading children on word learning tasks [149]. According to the authors, the results indicate that children with dyslexia have word learning difficulties beyond their phonological impairments. However, children with dyslexia were only impaired in the phonological aspects of word learning tests [150]. Other studies found that children with dyslexia performed worse than normal readers on phonemic awareness tasks and, to some extent, on rhyme awareness tasks, but they had only small impairments on verbal short-term memory tasks [151] (for review). Kimel et al. [152] found only a small difference between the performance of readers with DD and normal readers on tasks that required the children to learn repeated sequences of syllables. The authors conclude that if there is an impairment in serial order learning in children with dyslexia, it is mild and cannot be considered a core deficit. In agreement with these results, Debska et al. [153] found that the predominant deficit in children with DD was phonological (51%). A total of 26% of the children with dyslexia showed no cognitive deficit at all, and only 14% of them demonstrated an implicit learning impairment in a reaction time task. Children with dyslexia performed even better than normal readers on verbal learning tasks [154]. Lazzaro et al. [155] compared short-term and long-term memory in children with DD and normal readers. Children with DD performed worse than normal-reading, age-matched children but as well as normal-reading children at the same reading level on verbal, visual-object, and visual-spatial short-term and long-term memory tasks. The authors concluded that memory may not have a causal effect on reading performance.

Children with dyslexia improve when they receive a training that practices phonological awareness, rapid naming, phonemic decoding, word reading, spelling, and reading, e.g., [151,156,157,158,159,160]. Torgesen et al. [156] found that first graders with poor letter-sound knowledge who received an 8-month reading intervention in which the children practiced phonological awareness, rapid naming, phonemic decoding, word reading, spelling, and reading comprehension outperformed controls but only on the phonemic decoding, rapid naming, and spelling tasks. Reading accuracy and efficiency as well as spelling skills also improved when children with dyslexia received reading therapy [158]. Children with dyslexia improved their reading performance after taking part in a 16-week remediation program that included reading syllable lists as quickly as possible, phonological awareness exercises, memorizing syllables or words after they disappeared from the screen, and choosing real words from real and invented words [159]. After 6 weeks of computer-based training, in which children were taught to read single letters, letter combinations, and words of different lengths, reading ability improved only in boys but not in girls [160].

It is not surprising that phonological awareness, rapid naming, phonemic decoding, word reading, spelling, and reading comprehension improve after systematic training in these skills. When good readers read correctly, they learn which graphemes correspond to which phonemes, and each reading session can reinforce their knowledge of the grapheme–phoneme correspondence. Good reading requires a continuous learning process with reading practice. Poor readers, who read haltingly with many reading mistakes, do not have this constant reading experience. They realize that the words and sentences they are trying to read do not make sense and that their reading is therefore incorrect. They find reading unpleasant, so they avoid it. The ability to retrieve the correct sound sequences that correspond to letter sequences is a necessary condition for flawless reading. However, this is not the only necessary condition for flawless reading. Other necessary conditions include directing the gaze for a given time interval approximately to the middle of the word or word segment to be read, recognizing a string of letters that make up a word at a time, not starting the pronunciation too early, and performing eye movements which match the number of letters that can be recognized at a time. First graders learn letter–sound correspondences and how to replace letter-by-letter reading with reading a few letters simultaneously. These reading abilities are learned through the teacher’s reading instructions. Establishing other necessary conditions, such as where and how long to fixate in a word, how many letters to read simultaneously, and the appropriate eye movements, must be learned without teacher instruction through repeated reading in a self-generated learning process. Dyslexia develops when a child fails to generate at least one of these necessary conditions. Dyslexia can therefore be seen as an impaired ability to individually create all the necessary conditions for error-free reading. However, dyslexic children are not incapable of learning to create all the necessary and sufficient conditions for error-free reading. Repeated studies [20,21,146,147] have shown that children with dyslexia are able to learn to establish all the necessary and sufficient conditions in fewer than 30 min using computer-based reading therapy.

## 4. What Functional MRI and Cerebral Lesions Reveal about the Neurobiological Basis of Dyslexia

The question of whether an impairment is a cause of DD or whether it is merely an accompanying deficit with no causal significance is also fundamental to the assessment of neural dysfunctions that may occur together with impaired abilities such as dyslexia [161,162]. Theories regarding the neural basis of DD mainly rely on functional magnetic resonance imaging (fMRI) studies [125,126,127,128,129,163,164,165,166,167,168,169,170,171,172,173,174,175,176,177,178,179,180,181,182,183,184,185,186,187,188,189,190]. An increase in the blood-oxygen-level-dependent (BOLD) signal in an area of the brain shows that this area is receiving sensory afferents or input from other brain areas [191,192]. If the BOLD signal is smaller in dyslexic readers than in good readers, it does not mean that the activation of this brain area, such as the ventral occipitotemporal cortex including the word form area [163,165,167,169,179,181], is a necessary or sufficient condition for flawless reading. Underactivation of a brain area in dyslexic readers may be present in dyslexic readers without impacting the reading process. To conclude that an underactivated brain region contributes to reading impairment, a dysfunction in another brain area concomitant with the underactivated area must be excluded. If a brain region that plays a role in the reading process is functionally impaired, other brain regions may compensate for this impairment. It has been demonstrated that loss of one or even both occipital lobes in early development can be compensated to a considerable extent [193,194] and that reading is even possible in the absence of the left ventral occipito-temporal cortex [195]. It can also be assumed that not all functional impairments of neural networks are reflected in functional MRI [196].

Reading impairment may not be due to the underactivated but compensated region detected in functional MRI. It may be due to functional impairment of another region not sufficiently compensated. The results of many studies [163,197,198,199,200,201,202,203,204,205,206,207,208,209,210,211,212,213,214,215,216,217,218,219,220] indicate that brain regions of dyslexic readers that were less activated than those of normal readers contain the fusiform gyrus, superior temporal gyrus, precuneus, inferior parietal cortex, including the angular gyrus, and the inferior frontal gyrus of the left cerebral hemisphere. If at least one of these brain structures is underactivated in readers who are regarded as dyslexic according to commonly used reading tests, we may conclude that this underactivation is the cause of the reading disorder. However, this is not entirely correct. It has been repeatedly demonstrated [20,21,22,146,147] that readers with dyslexia need longer fixation intervals and longer verbal response times and that their reading saccades are unsuitable for fluent reading (Figure 1 and Figure 2). Repeated studies have also demonstrated that even children with severe dyslexia dramatically reduce the rate of reading mistakes within 30 min of practice when they adopt a new reading strategy tailored to their impaired abilities [20,21,22,146,147]. Children with dyslexia can learn (1) to fixate on the words or word segments to be read approximately in the middle, (2) to increase their fixation time, (3) not to attempt to recognize more letters simultaneously than they can recognize at a time, (4) to match the amplitudes of the saccades to the number of letters that they can read simultaneously, and (5) to increase the time from the onset of fixation of the words or word segments to be read to the onset of pronunciation. When dyslexic children used such a new reading strategy tailored to their abilities, the rate of reading mistakes was reduced to approximately one-third of the error rate before acquiring the new reading strategy. Reading was slowed down due to the necessary increase in fixation time and verbal response time, but the error rate was dramatically reduced. This means that the underactivation of cerebral regions observed in dyslexic readers causes impaired reading abilities only when the location of fixation, duration of the fixation interval, simultaneous recognition, saccades of eye movements, and verbal reaction times are not adapted to the reader. Activity in areas V1, V2, and V3, where the temporal summation is predominantly achieved and which is also present to a lesser extent in areas V4, the anteriorly adjacent area VO, area MT, and the intraparietal sulcus [119,125], precedes activity in the fusiform gyrus, superior temporal gyrus, precuneus, and inferior parietal cortex, including the angular gyrus and the inferior frontal gyrus of the left cerebral hemisphere. If areas V1, V2, and V3 need longer fixation times to complete temporal summation, insufficient temporal summation due to too short fixation times may cause lower activation in areas that receive input from areas V1, V2, and V3. This is in agreement with the finding that the fMRI signal of a cerebral region is mainly generated by input to this region [191,192]. It is also possible that a developmental dysfunction that affected the visual cortex also affected regions of the parietal and temporal lobe, whereby the latter dysfunctions did not substantially impair reading abilities.

It has been demonstrated [205,221] that surgical removal of the Visual Word Form Area in the left fusiform cortex results in an impaired ability to simultaneously recognize a string of letters. If only one specific cerebral damage occurred, if reading performance was normal before the cerebral damage and deteriorated after the cerebral damage, as was the case in these studies, this indicates that a normal function of the surgically removed brain structure is a necessary condition for normal reading performance. Therefore, one can conclude that the function of the Visual Word Form Area is also a necessary condition for the ability to simultaneously recognize a string of letters.

## 5. Conclusions

Whether there is a causal relationship between impairments that precede or coexist with dyslexia can only be assessed when based on a clear scientific concept of cause. A cause can be regarded as the absence of at least one necessary condition and/or the absence of all sufficient conditions. Whether a condition is necessary or sufficient can only be determined on the basis of experiments in which presumed necessary or sufficient conditions are alternately present or absent. Considering that correct reading requires the presence of all necessary conditions and at least one sufficient condition, dyslexia is present when at least one necessary condition or all sufficient conditions are missing. To test whether all the necessary and sufficient conditions for normal reading are met, it is necessary to find the conditions under which dyslexic children immediately and dramatically reduce their rate of reading errors without further training. This means that the search for necessary and sufficient conditions for reading includes a highly successful reading therapy. If one necessary condition or all sufficient conditions are lacking, this causes impaired reading performance.

The distribution of visual acuity in the retina requires eye movements to shift the word or word segment into the foveal region. The amplitudes of these eye movements must match the reader´s ability to simultaneously recognize a string of letters. To recognize a string of letters simultaneously, an individually required fixation time must be maintained to achieve the required temporal summation. Additionally, the individually required verbal reaction time must be maintained when reading aloud. When dyslexic readers learn a reading strategy in which all the necessary conditions are met, their reading performance improves immediately. This has enabled us to develop a powerful computer software that can improve the reading skills of children with severe dyslexia in a very short time. Repeated experiments with native German speakers have shown that phonological problems do not cause dyslexia in languages with high grapheme–phoneme correspondence.

Experiments to determine whether there is a causal relationship are often not feasible because it is impossible to set up experiments in which a necessary condition is alternately present or absent. The assumption of a causal relationship is then based on the finding that an impairment (e.g., poor reading skills) always occurs after or during an event (e.g., a brain lesion) and on what is already known about the function of a biological system (e.g., the brain).

Functional MRI and brain lesion studies have demonstrated that dyslexia results from uncompensated impairments of the primary and secondary visual cortex and areas that receive input from these early visual processing structures, such as the fusiform gyrus, superior temporal gyrus, precuneus, inferior parietal cortex, and left inferior frontal gyrus.

## Data Availability

No new data have been created.
